# Trust, trustworthiness and AI governance

**DOI:** 10.1038/s41598-024-71761-0

**Published:** 2024-09-05

**Authors:** Christian Lahusen, Martino Maggetti, Marija Slavkovik

**Affiliations:** 1 Department of Social Sciences, Universität Siegen, 57068 Siegen, Germany; 2 Université de Lausanne, Institute of Political Studies, CH-1015 Lausanne, Switzerland; 3 Information Science and Media Studies, Universitetet I Bergen, 5007 Bergen, Norway

**Keywords:** Computer science, Computational science

## Abstract

An emerging issue in AI alignment is the use of artificial intelligence (AI) by public authorities, and specifically the integration of algorithmic decision-making (ADM) into core state functions. In this context, the alignment of AI with the values related to the notions of trust and trustworthiness constitutes a particularly sensitive problem from a theoretical, empirical, and normative perspective. In this paper, we offer an interdisciplinary overview of the scholarship on trust in sociology, political science, and computer science anchored in artificial intelligence. On this basis, we argue that only a coherent and comprehensive interdisciplinary approach making sense of the different properties attributed to trust and trustworthiness can convey a proper understanding of complex *watchful trust* dynamics in a socio-technical context. Ensuring the trustworthiness of AI-Governance ultimately requires an understanding of how to combine trust-related values while addressing machines, humans and institutions at the same time. We offer a road-map of the steps that could be taken to address the challenges identified.

## Introduction

Artificial intelligence (AI) is now widely recognised as a socio-technical phenomenon that creates unprecedented opportunities while also possibly posing existential risks^1,2^. Overly positive or negative outlooks on the impact of AI tend to overestimate both the potential as well as the threat of artificial intelligence, but they are correct in highlighting the progressive incorporation of AI-based systems into all aspects of societal life^3^. This observation also applies to public authorities, which notably adopted AI because they see the merits of investing into technological innovation in order to increase the efficiency, effectiveness, and quality of services. Automation of decisions with the help of AI has thus been increasingly, and sometimes invisibly, introduced into core state activities such as public policy decision-making, internal management, and public service delivery in a wide range of areas^4,5^.

The relationships between public authorities and citizens are particularly affected by the growing reliance on AI automation, namely algorithmic decision-making (ADM)—software tools that use AI to aid and to make automated decisions. ADM is increasingly used to manage citizens’ applications and requests, identify personalized services, predict risks in regard to service provision, specify potential sanctions, and communicate decisions. ADM systems are thus having concrete effects in several domains, especially in—but not limited to—high-income countries. For instance, automation is being applied by public authorities to identify the eligibility for children’s allowances^6,7^, to select available employment offers for jobseekers^8^, to calculate social benefits^9^, to detect tax fraud^10,11^, and to forecast and monitor criminal behaviour^12,13^. The implications of these developments are considerable and need to be examined closely. In fact, public authorities are sole providers of service, particularly in sensitive areas of citizens’ lives, they have privileged access to personal data, take binding decisions, and might thus inescapably and directly affect beneficiaries and society as a whole.

The magnitude of the problem is determined by the fact that the growing insertion of AI into public service provision has ushered forms of AI-Governance, i.e., highly complex and dynamic socio-technical configurations that comprise technological, institutional, and regulatory elements. Specifically, they consist of:

– ADM applications developed for public authorities;
– Institutional practices that define the purposes and forms of usages; and
– A regulatory framework that both enables and constrains ADM deployment.

Algorithms introduce a strong transformative element into these configurations, because they constitute an inscrutable, opaque system whose operations, choices, and consequences are still poorly understood. They might encourage or facilitate improper or malicious use by individuals and governments, e.g., for criminal or censoring purposes. Furthermore, they may yield unpredictably inaccurate or biased results, as shown by the case of Dutch tax authorities who used a self-learning algorithm for spotting child care benefits fraud, which proved to be biased against lower incomes and ethnic minorities, and violated privacy laws^1^.

The question then is how aligned is AI-governance with the values of the society that it serves and that sustains it—particularly core democratic values? In this regard, trust and trustworthiness plays a particularly important role, because trust is at the same time a precondition, a product, and a foundational ethical value for functioning human societies. Our claim is that work on AI-governance “trust-alignment” is lacking a profound multi-disciplinary understanding of how trust and trustworthiness should be conceived and they operate in a socio-technical context where the main agent of trust and the main bearer of trustworthiness is not human but an algorithm.

In this paper, we propose to focus on AI-Governance as the main object of inquiry. We argue that the question of trust and trustworthiness can only be properly addressed, when considering the interplay between its three components: AI applications, administrative practices, and regulatory systems. Such a research object calls for an interdisciplinary research agenda that transcends the limited insights of previous studies. We argue that wide gaps exist in-between the different research fields when it comes to trustworthy AI. Only an interdisciplinary collaboration, involving sociology, political science, and artificial intelligence, is thus able to generate the necessary synergies to address the challenges associated with the development of value-aligned AI-Governance. The aim of this paper is thus to provide a selective overview of the core literature in research fields involved in the analysis of AI-Governance. It wishes to engage computational science, sociology and political science into an interdisciplinary dialogue aimed at merging available evidence and synchronising research agendas. For this purpose, the paper pursues the following goals: to (1) justify the relevance of a study of trust and trustworthiness of AI-Governance; (2) provide an overview of the state of the art in the three research fields; and (3) discuss implications for future research.

Our literature review is a systematic effort to merge interrelated disciplinary research fields. We started by identifying and defining the trustworthiness of AI-governance as the core research question, and then we engaged in a comprehensive bibliographic search, article screening and synthesis of findings for the three components of the socio-technical system^14^. The literature review is wide-ranging, but not exhaustive, because we are dealing with early accumulation of knowledge in highly dynamic research field, where trust-related issues are increasingly updated. Moreover, relevant evidence is unequally developed, with more consistent efforts in the fields of the social sciences, when compared to computational science. The aim of our overview is to provide a pluralistic account of the main issues at stake. Our contribution thus is to build a “point of departure” for scientists that will engage with the trust and trustworthiness of AI-government.

## Trust and trustworthiness

Finding the adequate definition of trust is an arduous undertaking, when considering the highly diverse interdisciplinary field relevant for the study of AI governance. A starting point for such a conceptualisation could be the proposal to understand trust as “the willingness of a party to be vulnerable to the actions of another party”^15^, be this other party another human, an institution or a machine. However, the agreement would only subsist under the condition that trust is related to human trustors, that is, when conceiving trust as a human property. Conceptual junctures emerge when considering that trust in humans, institutions and machines are categorically distinct. Additionally, it is necessary to remember that conceptual disagreements also run across disciplines, thus contributing to interdisciplinary discrepancies and disagreements. In fact, scholars diverge conceptually when relating trust to either behaviors, attitudes or relations^16–19^. Anchoring trust in behavioral practices and norms, in perceptions and preferences and/or in functional or formal properties of interactions between people, institution and/or machines has considerable implications for the way how trust is conceptualized, operationalized, and analyzed.

An interdisciplinary approach to the study of trust in AI governance should thus abstain from an overhasty synthesis of available evidence. This is also true for the focus of this collection, namely AI alignment to human and societal values. Trust is in itself a value, when defining a social value as an ethical conception of what is a desirable good within a given society. Scholars in various research fields would converge in this assessment, as trust is widely considered to be an important precondition and ingredient of societal cohesion, political stability, economic development, and/or technological innovation^20–24^.

However, societal values are intrinsically plural, dynamic and context-dependent, which means that even trust is a conditional posture. “We learn that, tentatively and conditionally, we can trust trust and distrust distrust, that it can be rewarding to behave as if we trusted even in unpromising situations”^25^. Unconditional trust can transcend what we conceive to be socially desirable, while conditional distrust might be a value in itself—when voiced through appropriate channels—because it may serve to prevent misbehaviour and keep social relations or societal systems in check^26–28^. Consequently, finding a careful balance between trust and distrust is crucial to ensure that trust is placed in those who deserve trust, whilst avoiding to place blind trust in the untrustworthy.

The alignment of AI to societal values has thus to consider the pluralism, dynamism and situatedness of values. This is particularly true with respect to the use of AI systems by public authorities, which are still opaque and largely unaccountable but potentially deploy effects erga omnes. In this regard, the number of relevant values increases, when considering the specificities of humans, institutions and machines as ‘trustworthy’ trustees. Scholars in the various research fields have engaged identifying those values that determine the attribution of trustworthiness, and the list of values that are considered to be relevant exhibit both, similarities and dissimilarities. In regard to interpersonal trust, for instance, it has been highlighted that competence, predictability, benevolence and integrity are crucial values that help to ascertain a trustee’s trustworthiness^29,30^. With respect to public institutions the list includes values pertinent for the qualities (e.g., competence, reliability, democratic participation), procedures (e.g., transparency, accountability) and results (e.g., effectiveness, general welfare, justice) of political-administrative work^31^. In regard to AI systems, a number of properties have been identified as trust-relevant, such as reliability, robustness, safety, interpretability, explainability, fairness, transparency, and accountability. The existence of partially overlapping but also varying lists and typologies of values demonstrates that AI governance as a socio-technical system has a series of distinct, yet interlocked problems of trustworthiness: there are doubts about the trustworthiness of processes and outputs of artificial intelligence, about the trustworthiness of humans and public authorities to properly handle this technology, and about the trustworthiness of governments to properly regulate this area of innovation. These problems of trustworthiness are, however, not necessarily identical, possibly complementary but maybe even incompatible. And this means that value alignment cannot rely on a simple list of shared values, but requires a careful analysis that helps to ascertain the specifics and interfaces between the various components of AI governance and their problems of trustworthiness.

Within computational science, the trustworthiness of AI is taken as a long-term goal and under that umbrella, in recognition of the complexity of the problem, several distinguished networks have been established. This, however, does not mean that computer scientists have explored the concept of trust and use this to guide the qualification and quantification of computational and interaction properties associated with trust and trustworthiness of AI. Topics studied with respect to trust and trustworthiness in the AI field pertain to issues as diverse as reliability, robustness, safety, security, bias, interpretability, privacy, explainability, fairness, transparency, resilience, and accountability^32,33^. For computer scientists it can be very hard to find a good point from which to learn about and engage with the conceptualisation of trust as studied in the social sciences, as this research is typically not part of Trustworthy AI agendas in computer science.

## Algorithmic decision-making (ADM), why trust matters

“Algorithmic decision-making” (ADM) is defined by the European parliamentary research service^34^ as computational systems that rely on the analysis of large amounts of personal data, usually using deep learning to infer correlations or, more generally, to derive information deemed useful to making decisions.

Deep learning re-energised neural network research in the early 2000s due to some fundamental research breakthroughs. Since then, Neural Networks (NN) have proved to be a useful tool for modelling complex phenomena. In the past decade, these networks have been at the core of widely diffused AI used for image recognition, natural language processing and, most recently, the generation of content^35^.

**Fig. 1:**
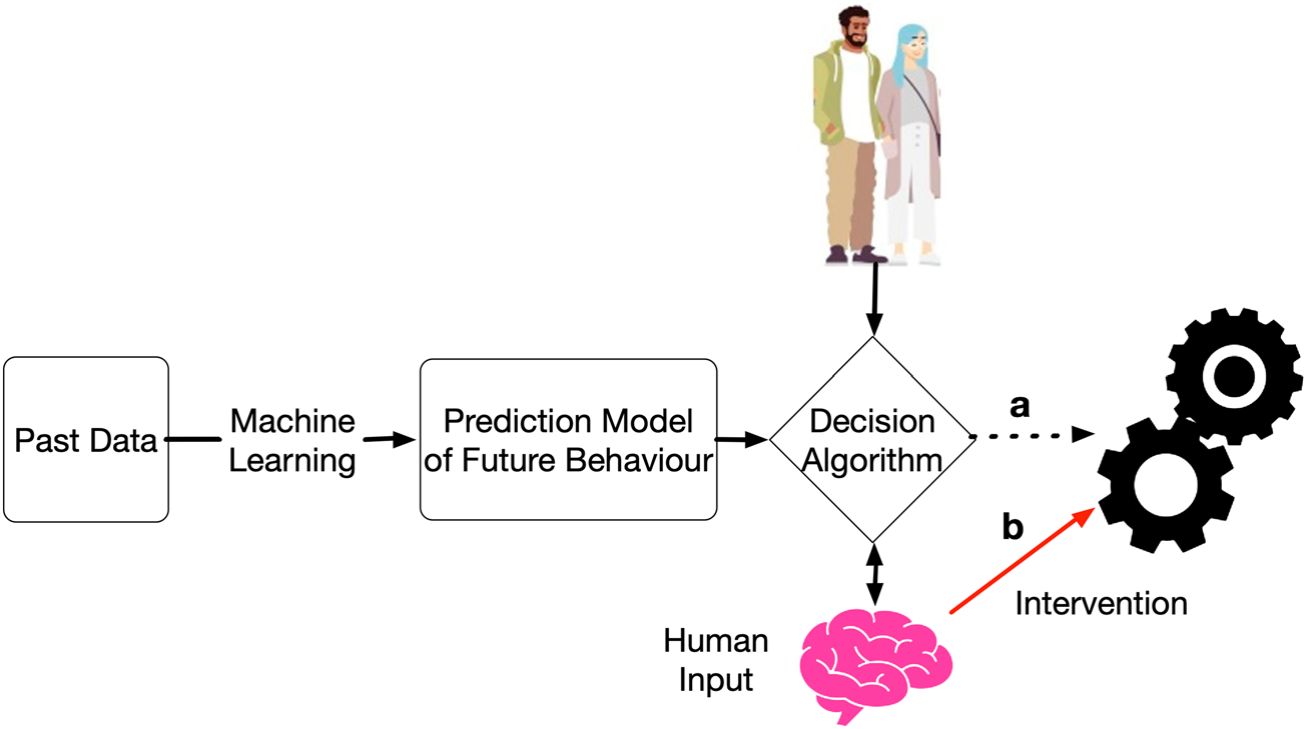
An algorithmic decision-making/-aiding system.

The ADM as it typically happens is illustrated in Fig. 1, where the decision instance is exemplified by two human figures, emphasizing that decisions are made about the people directly affected by them. The ADM system may be used to aid a human in making decisions, as shown by arrow b, by offering a comprehensive analysis of a large volume of data. Alternatively, the human level of intervention in the decision-making process may vary, as shown by arrow a, not being made by institution representatives but directly supplied to beneficiaries by the ADM system. Figure 1 uses a pink brain to illustrate human intervention in- or on-the-loop^36,37^ of the decision-making.

The European union general data protection regulation (GDPR)^38^ uses the term “automated decision-making” to refer to decisions being made without human involvement. Sometimes in the literature, both algorithmic and automated decision making are used as synonyms. For clarity, we should also state that we define an algorithm as “an unambiguous procedure to solve a problem or a class of problems. It is typically composed of a set of instructions or rules that take some input data and return outputs”^34^.

What separates NN artifacts from previous AI applications is their inbuilt incorrectness of output. Automated decisions that are obtained by utilising NN models can be assessed for accuracy, but unlike other non-AI computation, we do not have a mathematical guarantee of correctness. This is one of the main reasons why the intrinsic (or endogenous) trustworthiness of AI becomes such a dominant concern with the rising use of AI. Notwithstanding these limitations, the automation of tasks in the public sector using algorithmic decision-making is relatively widespread in public sector organisations^39–42^.

The use of ADM increasingly shapes core government functions, namely public service delivery, internal management, and policy decision making. In this regard, a number of challenges are often mentioned, including the variance in acceptance, privacy concerns, sample biases, discrimination, surveillance or the digital divide^43–47^. What is more, ADM implementation is complex and largely dependent on institutional practices and contextual factors^5^. These topics are considered to be urgent, because the automation of routines and decisions by public authorities deeply affect individuals, for instance, when they apply for social benefit, submit tax declarations, seek for health services or have violated the law. When a decision is made with the use of AI, public authorities insert the AI in the middle of the relationship between citizens and state institutions, making its (endogenous and exogenous) trustworthiness crucial. Due to accelerating technological developments, the trust relationship that holds between the institution and the individual needs to be re-built when including the AI system and its usage.

## An overview of individual disciplines

The insertion of ADM into the relationship between citizens and public authorities has aroused considerable research efforts^48–50^. Elements of these are devoted to trustworthiness issues and aim to settle a number of questions: Can AI become trustworthy and how? Can and should citizens and authorities trust a non-human agent and its usage by humans? Which forms of use by citizens and authorities are adequate? Which regulatory policies are in place, and which regulatory approach could be established by public authorities to fruitfully mediate adequate trust relationships unfolding between developers, adopters in the public sectors, and citizens as beneficiaries, with AI/ADM systems in the middle? The three disciplines, computer science, sociology, political science, have contributed considerably to these debates by providing evidence about the three pillars on which the topic resides (AI, society, political governance).

In the following sections we give an overview of the trustworthy AI literature in these three fields.

### Overview: citizens

The nexus between AI and society has become an increasingly important topic of analysis in the social sciences. Generally speaking, research has abandoned assumptions of binary relationships between distinct spheres (technology and society), acknowledging that AI is inherently embedded in social reality^51,52^. Scholars thus tend to speak of complex socio-technological systems that mirror established structures of social reality: machine-learning systems use informational and behavioural data generated by individual, corporate and state actors, who very often use decision-making models to replicate standard probabilistic reasoning widely used by humans, and the impact of machine-learning systems on society depends on the way ADM is used by humans or organisations^53^. Social sciences have thus painted a nuanced picture of the relation of AI and society, arguing that ADM is transforming but also reproducing societal reality^47^.

The topics of investigation are as diverse as the implications of artificial intelligence for social reality (e.g., work and leisure, news coverage and media usage, welfare services and public safety). But in regard to AI-governance, three topics are of paramount importance.

First, research has been interested in the perceptions and attitudes of citizens in regard to AI^48,54,55^. In spite of the categorical difference in trusting humans and machines, findings show an increasing readiness within society to assess AI in regard to trustworthiness, thus mirroring the growing relevance of AI-based applications in individual’s lives. Not only does trust vary depending on the respondents’ characteristics (e.g., age, gender, competence, education, personality traits, political orientations), but also regarding institutional and contextual factors (e.g., the countries’ socio-economic and cultural background)^55–59^. AI systems and the AI’s perceived characteristics (e.g., performance, reliability, anthropomorphism, rule-based operation) are crucial, as well^48,54^. Research thus suggests that citizens are moderately and conditionally trustful, which is considered positive, because blind trust in untrustworthy socio-technological systems is, in itself, highly problematic^60^.

A second strand of research centres more explicitly on the effects AI systems may have on the readiness of citizens to trust political institutions. Earlier studies of public trust in e-Government have already shown that the use of ICT and digitalisation can increase a citizen’s perception of institutional trustworthiness. However, trust remains highly conditional and depends on the general propensity to trust, the perception of the quality and usefulness of services, and risk sensitivity, particularly in regard to data privacy and security^61,62^. Digitalisation may also increase public perception of institutional efficiency and transparency, but not necessarily the recognition of institutional fairness, procedural or distributional justice^63^. The implementation of AI systems seems to exacerbate these conditionalities, because ADM systems are being adopted primarily for efficiency and effectivity reasons. At the same time, however, trust in the use of ADM by public authorities greatly depends on the specific areas of application^64^and the way they are implemented and used^65^.

A third research debate corroborates that conditionality of trust is an adequate public response to the application of ADM systems. ADM systems and their usage have been shown to generate problematic societal consequences, primarily by reproducing, even enlarging, existing social inequalities and power asymmetries^47,53,66^. This is particularly the case when ADM is used in contexts of public services, as has been shown in regard to algorithmic profiling in the context of public employment services^67^. Against this backdrop, issues of fairness and justice have received particular attention by social scientists when assessing ADM^68^. Before this backdrop, they (social scientists) were asked whether public acceptability of ADM systems might be influenced by fairness perceptions. Findings paint an inconclusive picture, as automated decision-making is evaluated on a par with human decision-making, sometimes even better^43,67,69,70^. Problems are associated with ADM design, but also with the institutional context of its applications, thus furthering discomfort, and in part, also contestation^71^.

### Overview: governance

In recent years, concerns about regulation and governance by and of algorithms have emerged as a major challenge for both scholars and policy makers. Public authorities—especially in, but not limited to, high-income countries-increasingly rely on AI and specifically on ADM systems to inform, assist, and make decisions; in that regard, for instance, OECD reports have documented the remarkable extent to which countries are integrating AI into public administration^72^. At the same time, governments also face urgent calls to regulate these systems^73–76^.

Increasing worries are indeed propagated by many social and political actors about a wide range of unwanted impacts of AI, and particularly of ADM, that threaten to erode essential collective goods, including democracy, solidarity, justice, privacy, and individual freedom^77,78^. At the same time, great expectations are formulated about the beneficial “disruptive” innovation potential of trustworthy ADM in countless areas, ranging from health to transportation and finance^45^ (see^79^ for a critique). In this sense, a trade-off may emerge between the efficiency gains associated with the deployment of AI and room for manoeuvre of public authorities to create and implement rules to protect the public good^80^. In that respect, key issues identified in the literature involve the need to ensure AI accountability by choosing transparent, interpretable models over black-box alternatives^81,82^ and the focus on governing the use, instead of the technology itself by discouraging and punishing the abuse of AI^83^. It has also been noted that unless specifically regulated, de facto rules by the private sector^84^ will govern the development and use of AI. Public regulation is nonetheless essential to ensure control over AI and, specifically, ADM systems and secure their trustworthiness^82,85–87^.

Different regulatory approaches are being developed, adopted, and tentatively implemented, all of which center on public trust^88,89^. The EU, in the wake of the AI Act, relies on comprehensive framework inspired by the precautionary principle, which imposes strict requirements on high-risk applications. The GDPR also has significant implications for AI, especially concerning data privacy and protection, aiming at ensuring that AI systems comply with stringent data handling and user consent requirements. For instance, in digital health, this does not only safeguards patient privacy but also builds public confidence in the use of AI for health purposes. The US approach, following the Blueprint for an AI Bill of Rights, is more fragmented and sector-specific, with different agencies overseeing AI use in various areas, while maintaining a conducive environment for innovation and technological advancement. The UK is developing a pro-innovation regulatory framework, whose goal is to position the country as a global AI leader, by relying on adaptive and principles-based regulation.

However, it is crucial to recognise that the current political and societal discourses on building trust in AI often fail to recognise that trust in AI is not unconditionally beneficial, and that focusing exclusively on how to build public trust in AI systems may obscure the questions about what might be required to actually build trustworthy systems^60^, namely because, as we have seen above, AI systems intrinsically operate in a state of limited trustworthiness. Rather than blind trust, a certain measure of functional distrust may indeed help to secure the long-term trustworthiness of AI and ADM systems, which bears the highest potential for transformative change, but also the highest risk of intrusiveness into people’s private life, especially when it is deployed by public authorities themselves. In this case, the regulation of algorithmic decision-making systems is particularly challenging, as public authorities take both the role of rule-maker and regulatory target^90^.

Given that trust is a relational property, the trustworthiness of AI depends not only on AI itself, but on its complex interplay with the main actors involved in the operation of AI systems, namely: developers, public authorities that adopt ADM systems, and citizens/users as beneficiaries. These complex interactions involve further dilemmas. Developers in the IT sector program, maintain and implement AI systems, and at the same time, their work can be both facilitated and challenged by AI. Public authorities (including regulators and intermediaries) face calls to regulate AI while already heavily relying on algorithmic systems to assist decision-making, and being confronted with a regulatory issue that is global in scale^91,92^. Citizens can be empowered by AI, and benefit from countless new services and opportunities, but biases, errors, new cleavages, social exclusion, surveillance and privacy protection concerns also loom large.

### Overview: computer science

The question that computer science is concerned with when it comes to trust and trustworthiness of AI in ADM specifically, is which technical tools and properties are relevant or should be developed in this context to make systems “endogenously” (or intrinsically) trustworthy.

What trusting a non-human agent means has been explored within the field of human-computer and human-robot interaction, see for e.g.^93,94^. People appear to have different expectations from machines compared to their expectations directed to other people, regardless of how intelligent those machines appear to be^93^. The trustworthiness that is attached to a non-human agent has a multidimensional structure that involves being capable, ethical, sincere, and reliable^32,95^.

Trust and trustworthiness have been extensively used in relation to Artificial Intelligence to signal the need for adequately embedding AI in a socio-technical society^96^. Chatila et al.^32^ ground trustworthiness of AI in general into delivery of service that can be justifiably trusted, and define it to be one that entails the following properties: availability, reliability, safety, confidentiality, integrity, maintainability, and security. Within each of these sub-topics, thousands of research papers with technical contributions are produced each year and published at computer science venues. However, although there is much research work under the keywords of trustworthy AI, not all of it discusses the subject^33^, making it difficult to ascertain where researchers are making progress.

Transparency is seen as quintessential to trust^97,98^, but at the same time, it is also recognised that transparency is not a “silver bullet”^2,99^ nor is it clear how it can be operationalised into more trustworthy AI. In the context of algorithmic decision-making, explainability, in particular, is a very powerful tool in facilitating trust in the process that involves AI^100^. The field of explainable artificial intelligence (XAI) is sometimes thought to subsume the work on transparency^59,101^ and sometimes to be a tool for transparency^98^. The field of explainable AI, specifically the moniker XAI, originates in a 2017 DARPA initiative^102^, with some very influential papers like^73^ beginning to appear in 2016. The field is thus very recent, but also very active. There are numerous systematic analyses already^59,103^ and even a systematic analysis of systematic analyses^104^ Work in XAI can range from developing technical tools like^105^ to meta discussions on what explainability should focus on, for example^106^.

Algorithmic decision-making is of particular concern to the field of algorithmic fairness, which specifically focuses on fairness in decisions made using machine learning. Algorithmic fairness is as new and as fruitful as XAI^4,107–109^. It studies problems of assessing the societal and individual impact of decisions made or aided by machine learning algorithms with the aim of ensuring that individuals (individual fairness) or protected groups (group fairness) are not discriminated against. Beyond assessment, algorithmic fairness is also concerned with developing methods to remove representational bias from data sets used to train prediction models, and methods that correct for allocational bias directly in the decision-making algorithms.

Necessarily, algorithmic fairness also studies possible trade-offs between a more efficient, more accurate and more “fair” decision algorithm. GDPR article 5^38^ requires that ‘personal data shall be: 1. processed lawfully, fairly and in a transparent manner in relation to the data subject (‘lawfulness, fairness and transparency’)”. Understandably, it would be practical to have a computational tool that automatically assesses the fairness of an ADM and the data used for it and by it. There are, at present, numerous proposed mathematical specifications of fairness^108,109^ that are not always grounded in the corresponding ethical desiderata^101,110^. Article 21 of the GDPR^38^ stipulates the right to object to processing of personal data, and the right to appeal a decision. The possibility for objection should be afforded by the computational design of the ADM^111–113^, and it is our task to create that affordance. Contestable AI is a very new field that studies how to make AI systems open and responsive to human intervention throughout their lifecycle, not only after an automated decision has been made^114^.

## Discussion

Dealing with the complexities of trust alignment for AI-governance requires joint efforts. On the one hand, the issue of trust is of paramount relevance with respect to the quick development of AI systems. AI-Governance is affected by substantial changes located at the level of technological advances, administrative usages and regulatory policies, whose proper functioning requires the presence of trust among the various actors involved, specifically including developers, administrative users, or regulators. Public administrators and regulators need to place trust in the ability of developers to generate trustworthy AI systems that are appropriate for the foreseen administrative usages and comply with regulatory goals and instruments. Similarly, developers need to trustfully rely on public authorities and regulators to properly employ, monitor and steer the technology in order to capitalize on its potentials while limiting downsides. For this purpose, it is important to develop sound scientific evidence to identify the necessary properties of AI systems, administrative usages, and regulations that sustain and enhance mutual trust, and to demonstrate how the features of trustworthy AI, usages and regulators are deeply intertwined. At the same time, interdisciplinary efforts are needed to determine the limits of trust and the conditions under which a watchful attitude could emerge and be maintained, as well as the mechanisms allowing for this vigilance to deploy. It is indeed only through the combination between trust and a watchful attitude (watchful trust), which implies a certain level of functional distrust, rather than blind trust, that developers, administrative users, and regulators can anticipate and tame potential risks and harms emanating from technological developments, administrative practices and regulatory measures. In particular, we identify four main challenges that require future interdisciplinary research efforts.

First, the mushrooming research efforts have contributed to a significant fragmentation of insights. There is an inflationary use of the concept of trust and trustworthiness^33^, and little theoretical clarity in regard to trust in AI, institutions and humans. This is complemented by a plethora of empirical measurements of trust in AI, institutions and humans, which makes almost impossible to systematically compare the evidence and produce cumulative knowledge.

Second, studies have provided insights into the acceptability of AI in general, and about the usage of ADM in institutional contexts. However, research is dominated by single case studies and lab experiments that say little about long-term dynamics and real-life settings, and are blind with regard to situational and contextual conditions. We thus know very little about whether and how ADM systems and practices contribute to the formation of trust and distrust. Additionally, the dominance of single case or country-specific analyses have led to inconclusive findings.

Thirdly, notwithstanding early attempts, which are fruitful but mainly set out to explore and scope the field^83,92^, research has yet to systematically develop an explicit research agenda addressing the regulatory challenges and responses to the technological transformations spurred by ADM systems and their use.

Fourth, research has not yet provided a consistent answer to the question of whether ADM systems and their utilisation can and should be trustworthy. There is an implicit agreement that blind trust is, generally speaking, inadequate posture in complex technological, social and political contexts, inasmuch as disconsolate distrust will be harmful.

In conclusion, to make substantial progress on the study AI value alignment, we need to first understand values, not only from the perspective of humans and human society, but also—and above all—from the perspective of machines, and how such values are intertwined and possibly interact.

## Conclusion

Considerable progress has been made in recent years in deepening our knowledge about trustworthy AI governance. This is due to the increasing societal and scientific relevance of AI, the growing need to provide robust knowledge, and intensifying research efforts in a number of research fields. However, we still see limits in our understanding of trust and the trustworthiness of AI governance.

We identify four challenges that require future interdisciplinary research efforts. “Interdisciplinary” is always desirable but not trivial to achieve. To overcome the fragmentation of insights (first challenge) we need to lower the barrier to understanding across researchers who study citizens, governance and AI on one end and those who “create” AI, on the other end. This may be accomplished by providing a simplified “interface” to a specific discipline: identifying the critical ideas, research programs, and accomplishments in each of the concerned research areas. This article is intended to be a “prototype” of such “interface” work.

A successfully discipline interfacing will enable empirical and theoretical framework of analysis that helps to identify adequate forms of trustworthy ADM systems, societal usages, and political regulations grounded on the concept of watchful trust. This work needs to be aligned with a systematic, comparative research agenda that empirically examines how the relevance of the trust and trustworthiness properties may vary, and how cross-sectional, longitudinal, and, respectively, contextual factors shape and condition different levels and forms of trust. Lastly, regulatory regimes are currently being developed from the perspective of regulation and governance of AI. This development needs to be aligned with and investigation of how they address the problem of AI trustworthiness (or fail to do so), and, in turn, how these embryonic regimes reshape trust and distrust in AI and in ADM.

In conclusion, to make substantial progress on the study of AI value alignment, we need to first understand values, not only from the perspective of humans and human society, but also—and above all—from the perspective of machines, and how such values are intertwined and possibly interact. Values that unequivocally require understanding from the perspective of machines are trust and trustworthiness. Progress in AI Governance depends on this understanding.

## Data Availability

This study does not use datasets. The articles datasets used and/or analyzed during the current study are available from the corresponding author upon reasonable request.
